# Genomic and transcriptomics analysis reveal putative secreted proteins expressed of *Pasteurella multocida* during 18β-glycyrrhetinic acid treatment

**DOI:** 10.3389/fvets.2024.1495924

**Published:** 2024-11-07

**Authors:** Zhongyuan Wu, Yuhan Zhang, Luyao Wang, Meng Mei, Yinsheng Qiu, Yu Liu, Shulin Fu, Jianglin Xiong, Qirong Lu, Pu Guo

**Affiliations:** ^1^Hubei Key Laboratory of Animal Nutrition and Feed Science, School of Animal Science and Nutritional Engineering, Wuhan Polytechnic University, Wuhan, China; ^2^State Key Laboratory of Biocatalysis and Enzyme Engineering, School of Life Sciences, Hubei University, Wuhan, China

**Keywords:** *P. multocida*, secreted proteins, transcriptomics analysis, *in silico* approach, eukaryotic-like domain

## Abstract

*Pasteurella multocida* is a gram-negative opportunistic pathogen that can infect both domestic animals and humans, leading to large economic losses to the livestock industry. 18β-Glycyrrhetinic acid, the main active component of *Glycyrrhiza glabra* L., has antibacterial properties. However, the virulence factors (especially the secreted proteins with eukaryotic-like domains) and pathogenesis of *P. multocida* and the regulatory effect of 18β-glycyrrhetinic acid have not been fully elucidated. This study focused on predicting secreted proteins with eukaryotic-like domains in *P. multocida* and examining the antibacterial effects of 18β-glycyrrhetinic acid on *P. multocida*. We combined transcriptomics analysis and *in silico* approaches to explore virulence factors in the *P. multocida* HB03 genome and identified 40 secreted proteins with eukaryotic-like domains regulated by 18β-glycyrrhetinic acid. Quantitative real-time polymerase chain reaction (qPCR) showed that compared with the *P. multocida* group, 18β-glycyrrhetinic acid significantly reduced the expression of aceF, gdhA, hpaG, and sel1L and increased the expression of galT and xynC, which was consistent with the transcriptomic data. Combining these qPCR results with the Gene Ontology and Kyoto Encyclopedia of Genes and Genomes annotation results showed that 18β-glycyrrhetinic acid interfered with bacterial energy metabolism and host interactions by regulating the expression of virulence factors in *P. multocida*. Moreover, molecular docking revealed that 18β-glycyrrhetinic acid had the potential to target aceF and hpaG, thus regulating the activity of secreted proteins. Our findings indicate that predicting the secreted proteins with eukaryotic-like domains in *P. multocida* and elucidating the regulatory effect of 18β-glycyrrhetinic acid provides a theoretical basis for the prevention and control of *P. multocida* infection and the development of alternative antibiotic therapies.

## 1 Introduction

*Pasteurella multocida* is a gram-negative opportunistic bacterial pathogen with a broad disease spectrum, and it can infect many wild and domestic animal species as well as humans (1–3). Because of its high morbidity and mortality in animals, this bacterium has caused serious economic losses to the livestock industry (4, 5). *P. multocida* strains are classified into five capsular genotypes (A, B, D, E and F) (6), and the *P. multocida* genome contains many genes encoding putative virulence factors. These include capsules, lipopolysaccharides, filamentous haemagglutinin adhesins, toxins, components of sialic acid metabolism, components of iron-sequestering systems, outer membrane proteins, siderophores, extracellular enzymes, and secreted proteins (2, 7, 8), all of which play important roles in host cell interaction and pathogenesis. Although *P. multocida* has been studied for more than 100 years, its virulence factors and pathogenesis remain unclear. An in-depth understanding and identification of the virulence factors of *P. multocida* are essential for developing new treatment and control strategies to combat infections in humans and animals.

Bacterial secreted proteins, which operate as virulence factors, play important roles in nutrient acquisition, adaptation, signal transduction, and virulence within bacteria and their infection of the host (9, 10). Moreover, eukaryotic-like proteins or proteins with eukaryotic-like domains in prokaryotes are a class of proteins found in prokaryotes, but they may have evolutionary origins in eukaryotes and have attracted increasing attention because of their potential involvement in mediating the interaction between microorganisms and hosts (11, 12). Current research on the pathogenicity of *P. multocida* mainly focuses on virulence factors such as outer membrane proteins, lipopolysaccharides, and capsule (7, 13–15). By comparison, research on the secreted proteins with eukaryotic-like domains in *P. multocida* is inadequate. Accurate prediction of bacterial secreted proteins is of great significance for annotation of the *P. multocida* genome as well as exploration of the molecular mechanisms underlying important biological phenotypes of *P. multocida*, such as bacterial virulence and drug resistance.

With the increasingly serious problem of antibiotic resistance, it is necessary to develop new antibiotic alternatives. Traditional drugs and natural products have incomparable advantages in the treatment of bacterial infections by regulating bacterial virulence factors (16, 17). 18β-Glycyrrhetinic acid, the main active component of *Glycyrrhiza glabra* L. (liquorice), has hepatoprotective, antiviral, anti-inflammatory, immunomodulatory, antioxidant, and antibacterial properties (18–20). The antibacterial effects of 18β-glycyrrhetinic acid against multiple pathogenic bacteria have been reported previously. For example, 18β-glycyrrhetinic acid inhibits the survival of methicillin-resistant *Staphylococcus aureus* and attenuates the expression of *saeR* and *hla* virulence genes (21). 18β-Glycyrrhetinic acid also exerts antimicrobial activity by inhibiting the mRNA expression of the haemolysis-related genes *hly* and *aerA* of *Aeromonas hydrophila* (22). Therefore, 18β-glycyrrhetinic acid can potentially be used as an antibacterial agent in clinical settings to promote the utilisation of high-quality *Glycyrrhiza glabra* L.

Our preliminary study showed that 18β-glycyrrhetinic acid could inhibit *P. multocida-*induced vascular endothelial inflammation (19). However, the antibacterial effect and underlying mechanisms of 18β-glycyrrhetinic acid on the virulence factors of *P. multocida* remain unclear. Thus, in the present study, we combined transcriptomics analysis and *in silico* approaches to explore virulence factors of the *P. multocida* HB03 genome, focusing particularly on eukaryotic-like secreted proteins. We then explored the potential of 18β-glycyrrhetinic acid in regulating *P. multocida* virulence factors at the levels of mRNA and protein by quantitative real-time polymerase chain reaction (qPCR) and molecular docking, respectively. Elucidation of these mechanisms and virulence factors will provide a theoretical basis for the prevention and control of *P. multocida* infection and the development of alternative antibiotic therapies.

## 2 Materials and methods

### 2.1 Bacterial culture and drug treatment

*P. multocida* HB03 was provided by Prof. Bin Wu (Huazhong Agricultural University, Wuhan, China) and cultured in Tryptose Soya Broth (Hopebio, Qingdao, China) with 5% (v/v) foetal bovine serum (Tianhang, Hangzhou, China) at 37°C. For analysis of the regulatory effect of 18β-glycyrrhetinic acid, 20 μg/mL 18β-glycyrrhetinic acid (MedChemExpress, Monmouth Junction, NJ, United States) was cultured with *P. multocida* HB03 for 10 h.

### 2.2 Secreted proteins of *Pasteurella multocida* HB03 genome screened by *in silico* approach

The *in silico* approach consisted of EffectiveELD and genome screening, which included prediction of Sec pathway secreted protein, Tat pathway secreted protein, and non-canonical pathway secreted protein.

For eukaryotic-like domain prediction, the FASTA format of *P. multocida* HB03 genomic protein was downloaded from the UniProt database^1^ and named HB03. To predict proteins containing eukaryotic-like domains using the EffectiveELD database through EffectiveDB,^2^ the FASTA file of HB03 was sent to job submission, and the submit button was clicked with default parameters.

For the prediction of Sec pathway secreted proteins, the N-terminal signal peptides of *P. multocida* HB03 genomic protein were identified using SignalP-5.0,^3^ classifying them into proteins recognized by type I and II signal peptidases. TMHMM-2.0^4^ was then used to predict the transmembrane structure of the protein sequences with the identified signal peptides, screening out proteins with either one or no transmembrane helices. Because the software could not accurately distinguish between the signal peptide and the transmembrane region when evaluating proteins with one transmembrane helix, it was necessary to use Phobius^5^ to re-predict these proteins. Proteins with a transmembrane region were then removed. For protein sequences without a transmembrane structure, PredGPI^6^ was used for glycosylphosphatidylinositol (GPI) anchor prediction analysis to screen out protein sequences containing GPI anchors. Finally, Cell-PLoc-2 software^7^ and PSORTb version 3.0.3 software^8^ were used to predict the subcellular localisation of the protein and retain the secreted protein. In summary, proteins with signal peptides, without a transmembrane helix, without GPI anchor sites, and the ability to be recognised by type I and II signal peptidases could be preliminarily predicted as Sec pathway secreted proteins.

For the prediction of Tat pathway secreted proteins, proteins with RR signal peptide and a *D* value greater than the default threshold of 0.36 were screened by TatP software.^9^ Proteins without a transmembrane helix structure were then identified using TMHMM-2.0 (see text footnote 4) and Phobius (see text footnote 5). These proteins were classified as Tat pathway secreted proteins.

For the prediction of non-canonical pathway secreted proteins, SignalP-5.0 (see text footnote 3) was used to screen proteins without signal peptides, and SecretomeP-2.0 software^10^ was then used to select sequences with secp values of >0.5. After further screening using MHMM-2.0 (see text footnote 4) and Phobius (see text footnote 5), the retained proteins with neither signal peptides nor transmembrane helices and with secp values of >0.5 were classified as non-canonical pathway secreted proteins.

To further screen for secreted proteins with eukaryotic-like domains, the results of EffectiveELD and genome screening were subjected to Venn diagram analysis of jvenn^11^ to identify secreted proteins with eukaryotic-like domains.

### 2.3 Transcriptomics analysis of *Pasteurella multocida* HB03

The bacterial samples were divided into two groups: a control group (*P. multocida* HB03, *n* = 3) and a treated group (*P. multocida* HB03 cultured with 20 μg/mL 18β-glycyrrhetinic acid, *n* = 3). The bacterial cells were harvested after 10 h of incubation and sent to Majorbio Bio-pharm Technology Co., Ltd. (Shanghai, China) for transcriptome sequencing. The RNA sequencing library was sequenced with Illumina NovaSeq 6000 (Illumina, San Diego, CA, United States). The data generated from the Illumina platform were analysed online using the Majorbio Cloud Platform.^12^ The differentially expressed genes were screened using DESeq2 software based on an absolute fold change of >1.5 with a *p*-value of <0.05.

### 2.4 Gene Ontology and Kyoto Encyclopedia of Genes and Genomes pathway annotations analysis of putative secreted proteins

The putative secreted proteins were identified using a Venn diagram tool by analysing the secreted proteins screened through the *in silico* approach and the differentially expressed genes identified by transcriptomics analysis. These putative secreted proteins were then mapped to terms in the Gene Ontology (GO) and Kyoto Encyclopedia of Genes and Genomes (KEGG) databases for functional and pathway analyses by the online Majorbio Cloud Platform (see text footnote 12).

### 2.5 qPCR

Total RNA of *P. multocida* HB03 was extracted using a bacterial RNA extraction kit (Vazyme, Nanjing, China) according to the manufacturer’s instructions. The 1 μg extracted total RNA was then immediately reverse-transcribed into cDNA using ABScript Neo RT Master Mix for qPCR with gDNA remover (ABclonal, Wuhan, China) according to the manufacturer’s instructions. The qPCR procedure was carried out using synthetic primers and BrightCycle Universal SYBR Green qPCR Mix with UDG (ABclonal, Wuhan, China) with a QuantStudio 1 Plus real-time PCR system (Thermo Fisher Scientific, Waltham, MA, United States). The 16S rRNA gene served as the reference gene, and the relative mRNA expression was calculated using the 2^−ΔΔCt^ method. The primers of all genes (Sangon, Wuhan, China) used in this study are shown in Table 1.

**Table 1:** Primers used in this study.

Gene name	Forward (5′–3′)	Reverse (5′–3′)
PMCN03_0121 (galT)	TAGCGGTGGTGCCTTATTGG	TAAAAGGCGCCGCATGAAAC
PMCN03_0319 (sel1L)	CAGACAGCACCAACTGCAAC	AGCTTGTGCCTGGTAATCCC
PMCN03_0914 (aceF)	CGGATATGCAAGGGGGTTGT	AATGAAACGTGCGCCATCTG
PMCN03_1217 (gdhA)	GTGGCTGAAGGGGCTAACAT	TTACCCGGACCAAAGAGCAC
PMCN03_1641 (xynC)	CTGGGGGATTAGTGCAGTCG	GGTGTAATGCTCGGTTTGGC
PMCN03_1741 (hpaG)	ACGCGTGAAAAATCGTGTGG	ACTCGCCATTGACCCATGTT
16S rRNA	GAATGTTGCGGTGAATAC	GGTTACCTTGTTACGACTTC

### 2.6 Molecular modelling methods

The protein structure of bacterial secreted proteins was constructed using SWISS-MODEL.^13^ Molecular docking between protein and chemical structures was performed with SYBYL-X 2.0 and visualised in three dimensions using PyMOL (23) and in two dimensions using LigPlot+ (24).

### 2.7 Statistical analysis

Statistical analysis was performed using SPSS 18 (SPSS Inc., Chicago, IL, United States). Data are expressed as mean with standard deviation. The significance of differences between two groups was evaluated by an unpaired two-tailed Student’s *t*-test. A *p*-value of <0.05 was considered as significant difference. A *p*-value of <0.01 was considered as extremely significant difference.

## 3 Results

### 3.1 Identification of putative secreted proteins of *Pasteurella multocida* HB03 by *in silico* approach and transcriptomics analysis

**Figure 1:**
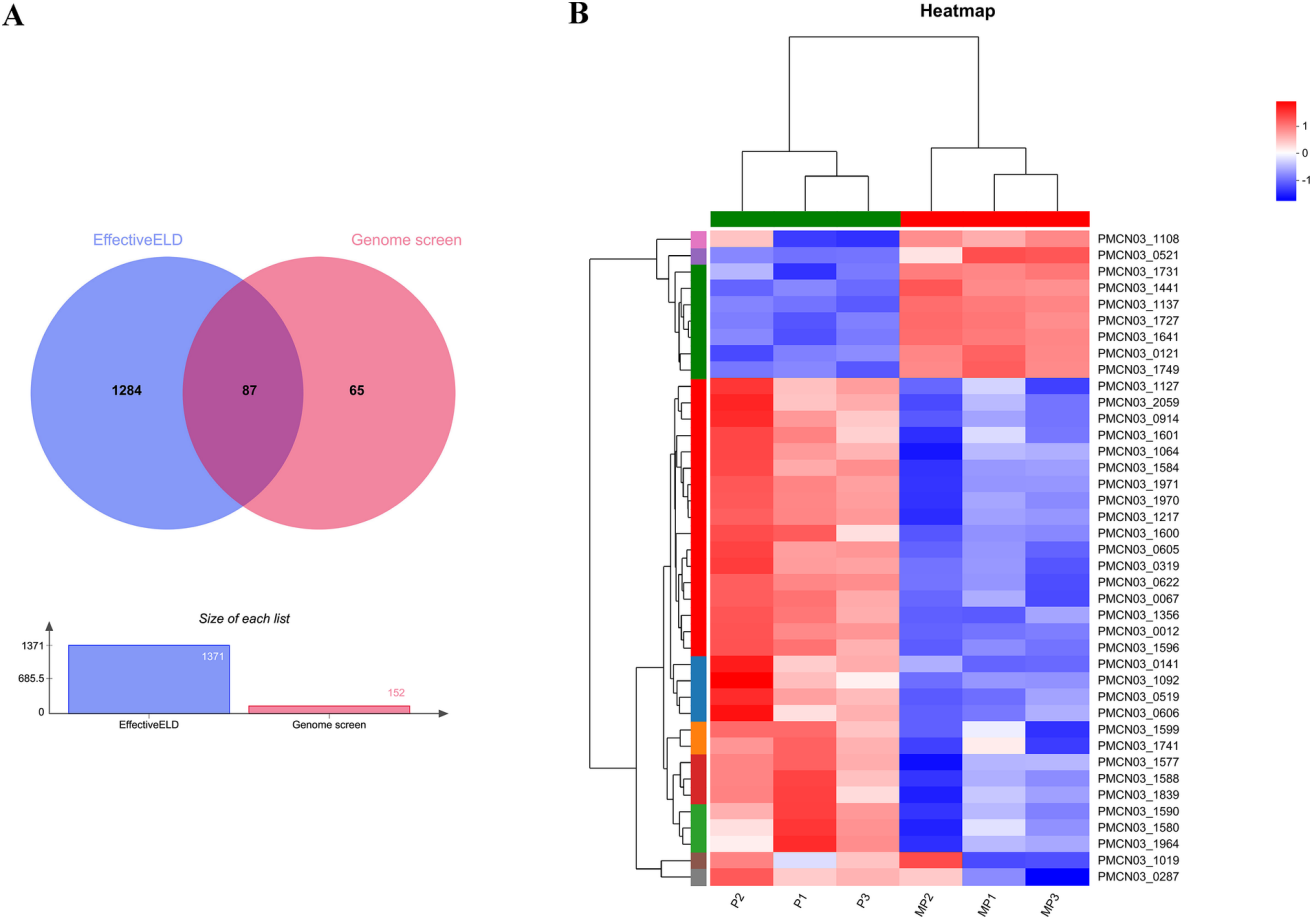
Putative secreted proteins of *P. multocida* HB03 were analysed by an *in silico* approach and transcriptomics analysis. (A) Venn diagram results of the putative secreted proteins of *P. multocida* HB03 analysed by EffectiveELD and genome screening. (B) Heatmap of 40 putative secreted proteins of *P. multocida* HB03 showed significant differences from the transcriptomics analysis.

The *in silico* approach and transcriptomics analysis were performed to screen the putative secreted proteins of the *P. multocida* HB03 genome. The *in silico* approach involved genome screening and EffectiveELD. Based on the genome screening, 152 secreted proteins were obtained including 28 secreted proteins of the Sec pathway, 7 secreted proteins of the Tat pathway, and 117 secreted proteins of the non-canonical pathway. In total, 1,371 proteins with eukaryotic-like domains were screened by EffectiveELD. A Venn diagram was produced using jvenn, and 87 secreted proteins were obtained (Figure 1A). To further explore the possible role of 18β-glycyrrhetinic acid in regulating secreted proteins, we focused on 87 secreted proteins identified using Venn analysis. From these, 40 putative secreted proteins of *P. multocida* HB03 that showed significant differences after 18β-glycyrrhetinic acid treatment were further screened from the transcriptomic analysis data (Figure 1B and Table 2).

**Table 2:** Forty putative secreted proteins of *P. multocida* HB03 genome.

Numbers	Putative proteins	Description
1	PMCN03_0012	50S ribosomal protein L33, rpl33
2	PMCN03_0067	DNA-binding protein Fis, Fis
3	PMCN03_0121	Galactose-1-phohate uridylyltransferase, galT
4	PMCN03_0141	Polysaccharide export protein Wza
5	PMCN03_0287	Hypothetical protein
6	PMCN03_0519	30S ribosomal protein S9, rps9
7	PMCN03_0521	Stringent starvation protein B, sspB
8	PMCN03_0605	50S ribosomal protein L35, rpl35
9	PMCN03_0606	50S ribosomal protein L20, rpl20
10	PMCN03_0622	Phohoribosylaminoimidazole carboxylase catalytic subunit, purE
11	PMCN03_0914	Dihydrolipoamide acetyltransferase, aceF
12	PMCN03_1019	50S ribosomal protein L31 type B, rpl31
13	PMCN03_1064	UDP-N-acetylglucosamine 1-carboxyvinyltransferase, murA
14	PMCN03_1092	Ribokinase, menG
15	PMCN03_1108	ftsI
16	PMCN03_1127	HflK protein, hflK
17	PMCN03_1137	Hypothetical protein
18	PMCN03_1217	Glutamate dehydrogenase, gdhA
19	PMCN03_1356	50S ribosomal protein L32, rpl32
20	PMCN03_1441	Anhydro-N-acetylmuramic acid kinase
21	PMCN03_1577	50S ribosomal protein L17, rpl17
22	PMCN03_1580	30S ribosomal protein S11, rps11
23	PMCN03_1584	50S ribosomal protein L15, rpl15
24	PMCN03_1588	50S ribosomal protein L6, rpl6
25	PMCN03_1590	50S ribosomal protein L14, rps14
26	PMCN03_1596	50S ribosomal protein L16, rpl16
27	PMCN03_1599	30S ribosomal protein S19, rps19
28	PMCN03_1600	50S ribosomal protein L2, rpl2
29	PMCN03_1601	50S ribosomal protein L23, rpl23
30	PMCN03_1727	Ribosomal RNA small subunit methyltransferase D
31	PMCN03_1731	Cell division protein FtsY, ftsY
32	PMCN03_1741	4-hydroxyphenylacetate degradation bifunctional isomerase/decarboxylase, hpaG
33	PMCN03_1749	ATP dependent phohoenolpyruvate carboxykinase, pckA
34	PMCN03_1839	Dihydroxy-acid dehydratase, ilvD
35	PMCN03_1964	30S ribosomal subunit protein S12, rps12
36	PMCN03_1970	50S ribosomal protein L1, rpl11
37	PMCN03_1971	50S ribosomal protein L1, rpl1
38	PMCN03_2059	30S ribosomal protein S20, rps20
39	PMCN03_0319	Sel1-like protein
40	PMCN03_1641	xynC protein

### 3.2 Functional annotations analysis of putative secreted proteins

**Figure 2:**
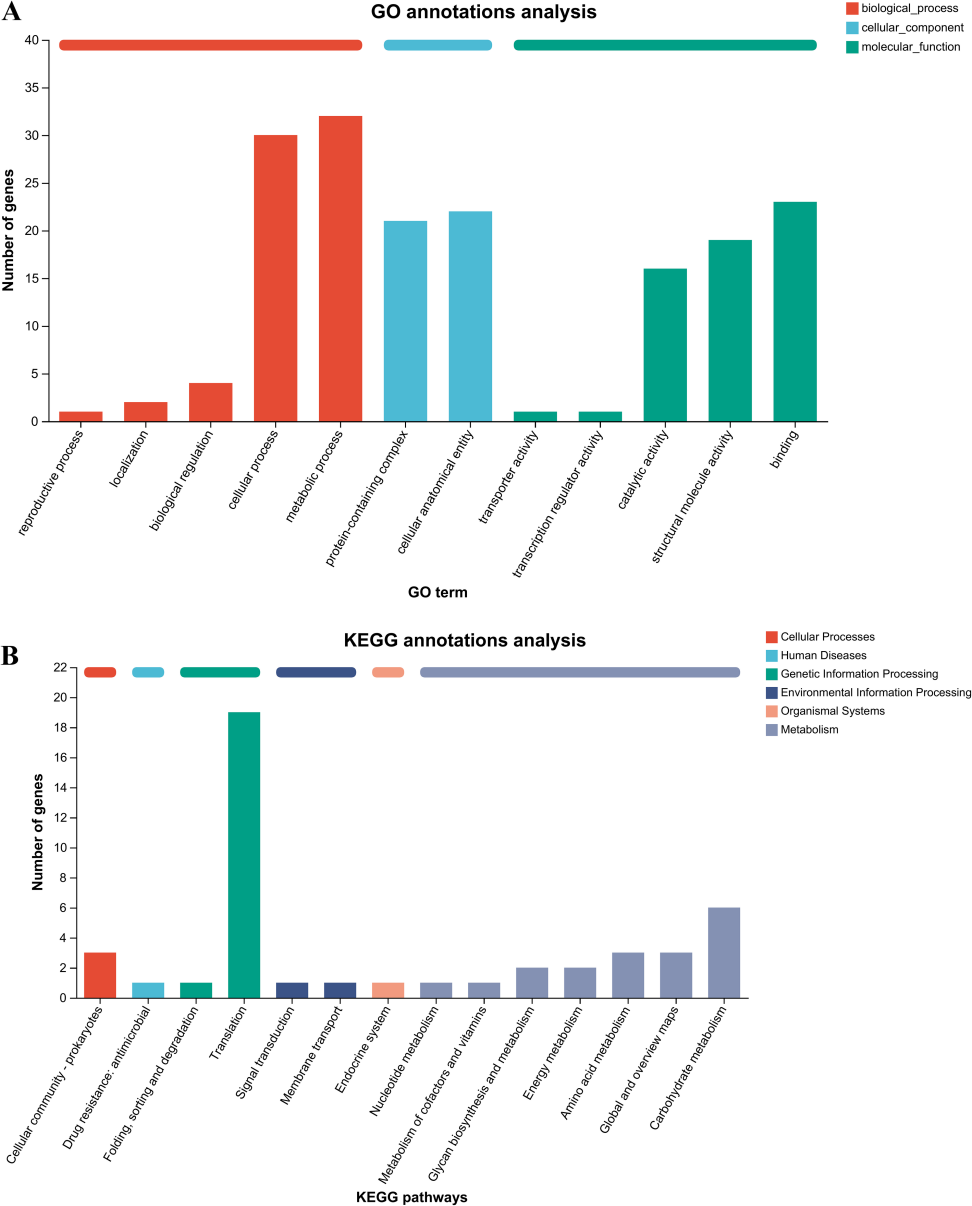
GO and KEGG annotations analyses of putative secreted proteins. (A) GO annotations analysis of 40 putative secreted proteins of *P. multocida* HB03. (B) KEGG annotations analysis of 40 putative secreted proteins of *P. multocida* HB03.

To classify the putative secreted proteins with a common function or biological pathway, it was necessary to obtain the functional annotation of these putative secreted proteins by GO and KEGG pathway enrichment analyses. The top 12 significantly enriched items from the GO analysis are shown in Figure 2A. The entries related to biological processes mainly included metabolic processes, cellular processes, biological regulation, localisation, and reproductive processes. The entries regarding cellular components mainly included cellular anatomical entities and protein-containing complexes. The entries related to molecular function mainly included binding, structural molecule activity, catalytic activity, transcription regulator activity, and transporter activity. The KEGG annotations analysis showed that the secreted protein-related pathways were mainly enriched in translation, signal transduction, cellular community-prokaryotes, drug resistance, membrane transport, the endocrine system, carbohydrate metabolism, amino acid metabolism, energy metabolism, and glycan biosynthesis and metabolism (Figure 2B).

### 3.3 18β-Glycyrrhetinic acid regulates the gene expression of secreted proteins encoded by *Pasteurella multocida* HB03

**Figure 3:**
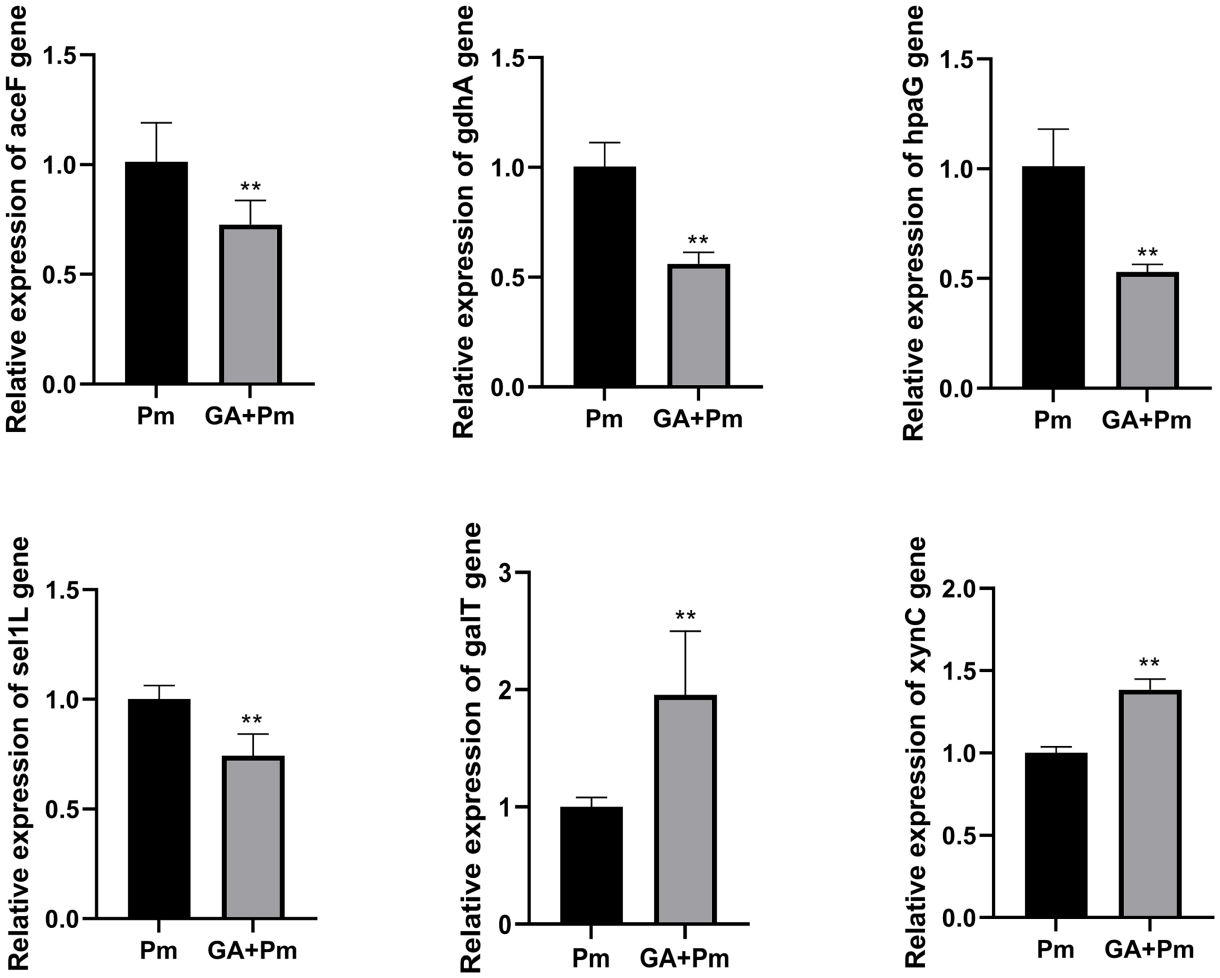
qPCR results of 18β-glycyrrhetinic acid regulation of gene expression of proteins secreted by *P. multocida* HB03. Compared with the *P. multocida* group, 20 μg/mL 18β-glycyrrhetinic acid significantly reduced the expression of *aceF*, *gdhA*, *hpaG*, and *sel1L* and increased the expression of *galT* and *xynC*. Pm, *P. multocida*; GA + Pm, *P. multocida* group treated with 20 μg/mL 18β-glycyrrhetinic acid. *p* < 0.01 (^**^) was defined as extremely significant difference.

Based on the results of the functional annotations analysis, the following secreted proteins related to energy metabolism, carbohydrate metabolism, cellular community, and transporter activity were selected: PMCN03_0121 (*galT*), PMCN03_0319 (Sel1-like protein, termed *sel1L*), PMCN03_0914 (*aceF*), PMCN03_1217 (*gdhA*), PMCN03_1641 (*xynC*), and PMCN03_1741 (*hpaG*). The qPCR results showed that compared with the *P. multocida* group, 20 μg/mL 18β-glycyrrhetinic acid significantly decreased the expression of *aceF*, *gdhA*, *hpaG*, and *sel1L* and increased the expression of *galT* and *xynC* (Figure 3), which was consistent with the transcriptomic data. The above results validated the accuracy of the transcriptomic data and indicated that 18β-glycyrrhetinic acid can effectively regulate the gene expression of secreted proteins. This suggests its potential as a candidate drug for the prevention and control of *P. multocida* infection.

### 3.4 Molecular docking analysis of 18β-glycyrrhetinic acid targeting secreted proteins of *Pasteurella multocida* HB03

**Figure 4:**
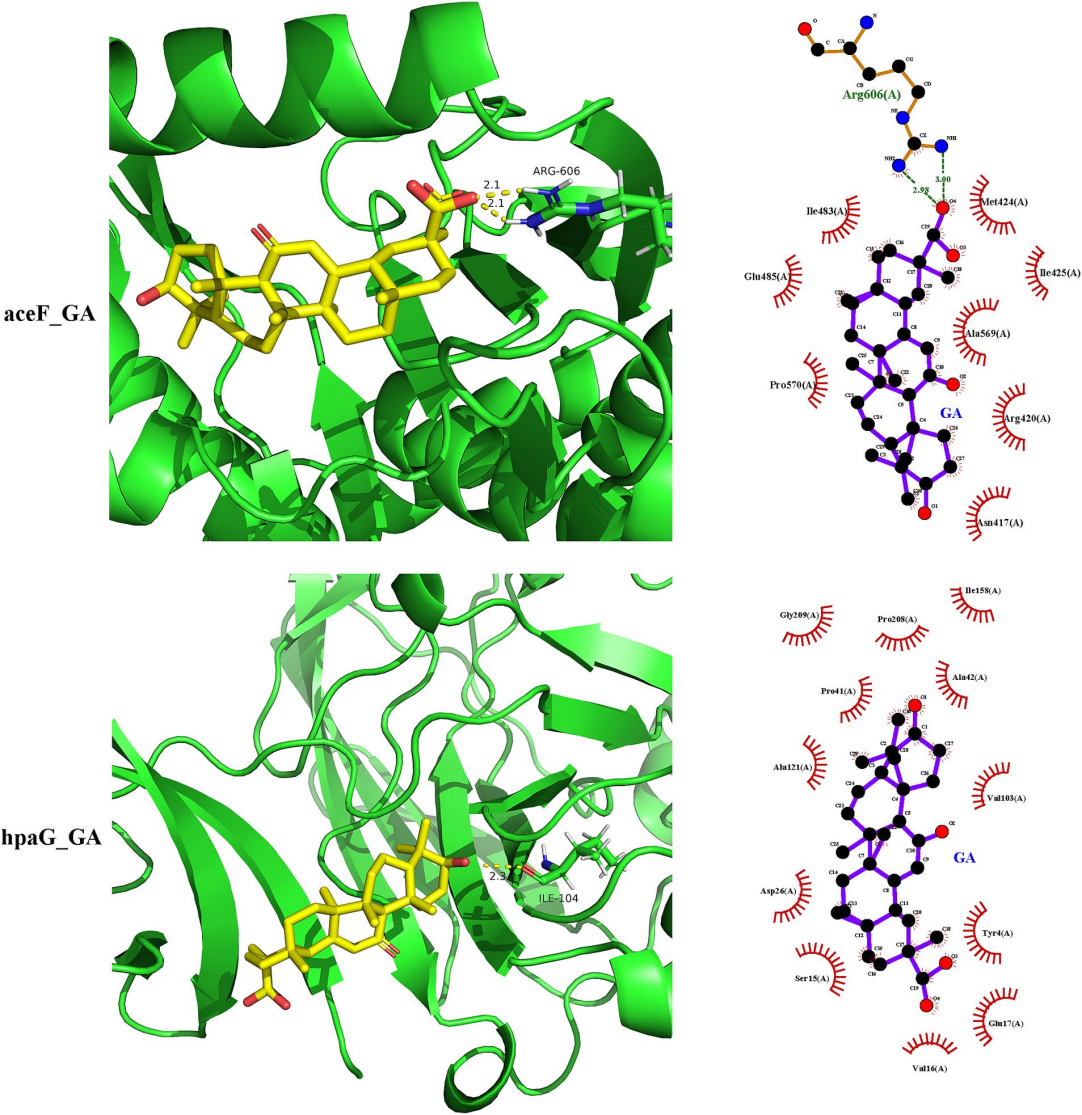
Molecular docking results of 18β-glycyrrhetinic acid targeting secreted proteins of *P. multocida* HB03. 18β-Glycyrrhetinic acid has the potential to target *aceF* and *hpaG*. The upper part of the figure shows the docking of *aceF* and 18β-glycyrrhetinic acid analysed by SYBYL-X 2.0, visualised by PyMOL (left image) and LigPlot+ (right image).

The functional annotation analysis of the putative secreted proteins showed that they possessed both structural molecule activity and catalytic activity. Based on these results, *galT*, *sel1L*, *aceF*, *gdhA*, *xynC*, and *hpaG* were selected as receptors for binding ability prediction with 18β-glycyrrhetinic acid. The docking score indicated the binding potential between the ligand and the receptor. A docking score of >4 suggested binding potential, with higher scores indicating greater stability. The docking score for binding of 18β-glycyrrhetinic acid with each target shown in Table 3, and the visualisation results are shown in Figure 4. The results indicated that 18β-glycyrrhetinic acid has the potential to target *aceF* and *hpaG*. The above molecular docking results further validated the accuracy of the results of the transcriptomics analysis and *in silico* approach.

**Table 3:** Docking score of 18β-glycyrrhetinic acid with each target.

Protein	Ligand	Docking score
aceF	18β-glycyrrhetinic acid	5.3691
hpaG	18β-glycyrrhetinic acid	5.3108
xynC	18β-glycyrrhetinic acid	3.9993
gdhA	18β-glycyrrhetinic acid	3.4069
galT	18β-glycyrrhetinic acid	3.0980
sel1L	18β-glycyrrhetinic acid	1.8211

## 4 Discussion

*P. multocida* is a gram-negative opportunistic pathogen that can infect many wild and domestic animal species as well as humans, leading to significant economic losses to the livestock industry (25). Numerous studies have shown that bacterial virulence factors play an important role in the pathogenicity of *P. multocida*, but the virulence factors of secreted proteins with eukaryotic-like domains have not been fully studied. Moreover, natural products can play an antibacterial role by regulating bacterial virulence factors (17). Therefore, it is of great clinical significance to study the virulence factors of secreted proteins with eukaryotic-like domains in *P. multocida* and the regulatory effects of natural products. In the present study, we combined transcriptomics and *in silico* approaches to explore virulence factors of the *P. multocida* HB03 genome, identifying 40 secreted proteins with eukaryotic-like domains regulated by 18β-glycyrrhetinic acid. Further qPCR revealed that 18β-glycyrrhetinic acid indeed regulated the mRNA expression of these secreted proteins, and molecular docking revealed that 18β-glycyrrhetinic acid had the potential for targeted regulation of the activity of the secreted proteins. Predicting secreted proteins with eukaryotic-like domains in *P. multocida* and elucidating the regulatory effect of 18β-glycyrrhetinic acid will provide a theoretical basis for the prevention and control of *P. multocida* infection and the development of alternative antibiotic therapies.

As virulence factors, bacterial secreted proteins play an important role in bacterial pathogenicity and mediating the interactions between the bacteria and host. Thus, to further clarify the pathogenicity of *P. multocida*, it is particularly important to predict its secreted proteins. In a previous study, 50 putative secreted proteins of high- and low-virulent bovine *P. multocida* were found by data-independent acquisition liquid chromatography tandem mass spectrometry combined with bioinformatics analysis (Sec pathway secreted protein prediction, Tat pathway secreted protein prediction, and non-canonical pathway secreted protein prediction) (9). In the present study, the genome screening process revealed 152 secreted proteins, including 28 secreted proteins of the Sec pathway, 7 secreted proteins of the Tat pathway, and 117 secreted proteins of the non-canonical pathway. Notably, bacterial proteins with eukaryotic-like domains serve as key players in host–pathogen interactions (26). EffectiveDB was employed to predict eukaryotic-like proteins in the whole genome of *Mycobacterium tuberculosis*, revealing that protein phosphatase PtpB serves as secreted protein with eukaryotic-like domains in *M. tuberculosis*. PtpB inhibits pyroptosis and counteracts host immunity (27). Based on this principle, we applied EffectiveELD software of EffectiveDB to screen the genome of *P. multocida* HB03 and identified 1,371 proteins with eukaryotic-like domains. In addition, a Venn diagram was applied to further screen the results of the genome screening and EffectiveELD prediction, resulting in a final 87 secreted proteins containing eukaryotic-like domains. Transcriptomic analysis of high- and low-virulence bovine *P. multocida* previously showed that a large number of virulence-related differentially expressed genes might be responsible for the virulence of *P. multocida* (28). Based on the 87 secreted proteins identified through Venn analysis, 40 putative secreted proteins of *P. multocida* HB03 that showed significant differences after 18β-glycyrrhetinic acid treatment were further screened from the transcriptomic analysis data. Thus, the present study explored the whole-genome sequences and transcriptome expression profiles related to virulence factors of secreted proteins containing eukaryotic-like domains in *P. multocida* HB03 as well as how these are regulated by 18β-glycyrrhetinic acid.

GO and KEGG pathway annotations are effective methods to evaluate the function of *P. multocida* virulence factors (29). Zhan et al. (14), performed functional enrichment analysis of filtered virulence-related differentially expressed genes in a natural isolate fluoroquinolone-sensitive strain (Pm3) and a highly fluoroquinolone-resistant strain (Pm64). The authors found that differentially expressed genes were enriched in kinases and certain molecules’ binding abilities within GO enriched items, as well as in the citrate cycle, amino acid biosynthesis and metabolism, glutathione metabolism, and fatty acid biosynthesis and metabolism. This indicates that controlling enzyme activity related to virulence factors or the expression of virulence factors related to energy metabolism can help reduce antibiotic resistance. Genomic characterisation of *P. multocida* HB01 and SHZ01 showed that the GO and KEGG pathway annotations of virulence factors involved in glycolysis/gluconeogenesis and carbohydrate metabolism as well as the presence of an intact TCA cycle are helpful for understanding the pathogenesis and genetic characteristics of *P. multocida* (29, 30). The transcriptional results of *P. multocida* to three classes of antibiotics with a sub-minimum inhibitory concentration (1/4 MIC) (amoxicillin, chlortetracycline, and enrofloxacin) showed that differentially expressed virulence factors were enriched in carbohydrate transport and metabolism, signal transduction mechanisms, amino acid transport and metabolism, and energy production and conversion. This analysis also elucidated the molecular basis underlying the therapeutic efficacy of antibiotics with 1/4 MIC (31). The minimum inhibitory concentration of 18β-glycyrrhetinic acid against *P. multocida* is 1,024 μg/mL (19). Moreover, in the present study, the GO and KEGG pathway annotations showed that 18β-glycyrrhetinic acid at concentrations below 1/4 MIC mainly regulated the expression of 40 putative secreted proteins with eukaryotic-like domains by regulating pathways involved in structural molecule activity, catalytic activity, signal transduction, cellular community-prokaryotes, drug resistance, carbohydrate metabolism, amino acid metabolism, energy metabolism, and glycan biosynthesis and metabolism. These findings indicate that intervening in the energy metabolism of bacteria and the structural molecule activity of secreted proteins can help alleviate the pathogenic effect of secreted proteins.

Numerous metabolic processes and functions associated with virulence factors are responsible for the pathogenic effects of bacteria. These include carbohydrate metabolism, energy metabolism, structural molecule activity, catalytic activity, signal transduction, cellular community-prokaryotes, and glycan biosynthesis and metabolism. With respect to virulence factors of energy metabolism, 4-hydroxyphenylacetate degradation bifunctional isomerase/decarboxylase (*hpaG*) is involved in the metabolism of aromatic compounds and the degradation of 4-hydroxyphenylacetate. This process further affects the preparation of pyruvate and succinate semialdehyde from 4-hydroxyphenylacetate (32). Additionally, the acetyltransferase component of pyruvate dehydrogenase complex (*aceF*) transfers acetyl to CoA, forming acetyl-CoA, which plays a role in central carbon metabolism and energy metabolism (30, 33, 34). Moreover, glutamate dehydrogenase (*gdhA*) plays a role in amino acid biosynthesis, catalysing the reversible deamination of L-glutamate to α-ketoglutarate, which is also the main link in carbon and nitrogen metabolism (35). These findings indicate that *hpaG*, *aceF*, and *gdhA* may affect pyruvate and α-ketoglutarate synthesis, thus further affecting the TCA cycle, amino acid biosynthesis, and energy metabolism. The present study showed that 18β-glycyrrhetinic acid inhibited the gene expression of *hpaG*, *aceF*, and *gdhA*, thus exerting a regulatory effect on the pathogenicity of *P. multocida*. However, 18β-glycyrrhetinic acid also increased the expression of *xynC* and *galT*. Acetyl esterase (*xynC*) is involved in carbohydrate metabolism, energy metabolism, and electron transport (36). Galactose-1-phosphate uridylyltransferase (*galT*) participates in galactose metabolism by catalysing the reversible conversion of glucose-1-phosphate and UDP-galactose to galactose-1-phosphate and UDP-glucose (37, 38). The change in the expression level of *xynC* and *galT* may be a compensatory effect of energy metabolism in *P. multocida*, but it is worth further studying how 18β-glycyrrhetinic acid increases the expression of these genes, thereby exerting its role in resisting bacterial pathogenicity. Regarding virulence factors of signal transduction and cellular community-prokaryote, sel1-like repeat proteins may establish a link between signal transduction pathways in eukaryotes and bacteria (39). This may not only influence the expression of genes involving in the type III secretion system but may also contribute to the pathogen’s ability to infect the host (40). 18β-Glycyrrhetinic acid may exert its resistance to bacterial pathogenicity by reducing the expression of *sel1L*. With regard to structural molecule activity and catalytic activity, the molecular docking results showed that 18β-glycyrrhetinic acid may inhibit the activity of secreted proteins with eukaryotic-like domains, including those encoded by the genes *aceF* and *hpaG*, and then play a role in inhibiting bacterial pathogenicity. However, its in-depth mechanism of action remains to be further explored. The above studies indicate that 18β-glycyrrhetinic acid can interfere with bacterial energy metabolism and host interactions by regulating the expression of virulence factors in *P. multocida*. However, combining the prediction of secreted proteins with eukaryotic-like domains and the regulatory effect of 18β-glycyrrhetinic acid on virulence factors, it is worth further studying how 18β-glycyrrhetinic acid interferes with the interaction between bacteria and hosts through these secreted proteins.

Some limitations exist in the current study. Although the secreted proteins of *P. multocida* HB03 were predicted based on the transcriptomics and *in silico* analysis in this manuscript, but the regulatory effect of 18β-glycyrrhetinic acid on those secreted proteins still need to further investigated in both *in vitro* and *in vivo* models. In addition, although 18β-glycyrrhetinic acid downregulated the gene expression of *aceF* and *hpaG*, and molecular docking results showed potential binding potential between aceF or hpaG and 18β-glycyrrhetinic acid, further experiments are needed to confirm the potential of *aceF* and *hpaG* as virulence factors in *P. multocida* and the molecular docking results.

In summary, the combined transcriptomics analysis and *in silico* approaches in this study uncovered the putative secreted proteins of the *P. multocida* HB03 genome and the regulatory effects of 18β-glycyrrhetinic acid. 18β-Glycyrrhetinic acid can interfere with bacterial energy metabolism and host interactions, and it can inhibit the activity of secreted proteins with eukaryotic-like domains, thus exerting an antibacterial effect on *P. multocida*. These comprehensive analyses improve our understanding of the molecular pathogenicity of *P. multocida* and will contribute to the development of natural products against this pathogen.

## Data Availability

The original contributions presented in the study are included in the article/supplementary material, further inquiries can be directed to the corresponding authors.

## Footnotes

^1^ https://www.uniprot.org/

^2^ https://www.effectivedb.org/

^3^ https://services.healthtech.dtu.dk/services/SignalP-5.0/

^4^ https://services.healthtech.dtu.dk/services/TMHMM-2.0/

^5^ https://phobius.sbc.su.se/

^6^ https://busca.biocomp.unibo.it/predgpi/

^7^ http://www.csbio.sjtu.edu.cn/bioinf/Cell-PLoc-2/

^8^ https://www.psort.org/psortb/

^9^ https://services.healthtech.dtu.dk/services/TatP-1.0/

^10^ https://services.healthtech.dtu.dk/services/SecretomeP-2.0/

^11^ https://www.bioinformatics.com.cn/static/others/jvenn/example.html

^12^ www.Majorbio.com

^13^ https://swissmodel.expasy.org/
